# Case report: Conservative treatment of an intraperitoneal bladder rupture

**DOI:** 10.1016/j.radcr.2024.11.089

**Published:** 2024-12-20

**Authors:** Noah J. Sandel, Matthijs Duijn, Liselotte M.S. Boevé

**Affiliations:** a Department of Urology, OLVG, Amsterdam, The Netherlands; b Amsterdam University Medical Centers, University of Amsterdam, Department of Urology, Amsterdam, The Netherlands

**Keywords:** Bladder rupture, Bladder perforation, Conservative treatment, Extraperitoneal, Intraperitoneal

## Abstract

Bladder ruptures are uncommon but potentially life-threatening conditions, that necessitates prompt medical intervention. Urological trauma guidelines differentiate between intraperitoneal and extraperitoneal bladder ruptures. Intraperitoneal bladder ruptures typically require surgical repair, while extraperitoneal bladder ruptures are often treated conservatively. This case report presents a 25-year-old male patient with a significant intraperitoneal bladder rupture, successfully treated through conservative management. This case highlights the potential for nonoperative treatment in selected patients with intraperitoneal bladder injury, contributing to the evolving discussion on the management of urological trauma.

## Introduction

Bladder ruptures are relatively uncommon but potentially life-threatening conditions, where prompt medical response is essential. Most cases of bladder rupture are associated with high-impact trauma and frequently occurring in conjunction with pelvic fractures [1]. However, bladder ruptures can also result from less severe trauma, particularly when the bladder is full and distended. Sudden increases in intravesical pressure, such as from minor impact, can cause a distended bladder to rupture, especially at the bladder dome, which is more vulnerable due to its anatomical position and thinner wall.

Common risk factors that increase the likelihood of bladder ruptures include prior radiotherapy, neurogenic bladder, recurrent cystitis, alcohol intoxication, urinary retention, and bladder malignancies. Symptoms of bladder rupture commonly include hematuria, along with abdominal pain, nausea, vomiting, and difficulty urinating [2].

To assess treatment, urological trauma guidelines classify bladder ruptures based on the anatomical location of the injury: intraperitoneal, extraperitoneal and combined intraextraperitoneal. In general, these guidelines recommend conservative treatment for minor extraperitoneal bladder ruptures, whereas larger extraperitoneal or intraperitoneal ruptures are preferably treated surgically. Conservative treatment often involves placement of a transurethral Foley catheter combined with antibiotic prophylaxis [[3], [4], [5]].

In recent years, there has been an increased tendency towards treating intraperitoneal bladder ruptures conservatively [6,7]. This case report presents a rare instance of a large intraperitoneal bladder rupture in a healthy 25-year-old male, successfully managed by conservative treatment.

## Case presentation

**Fig. 1:**
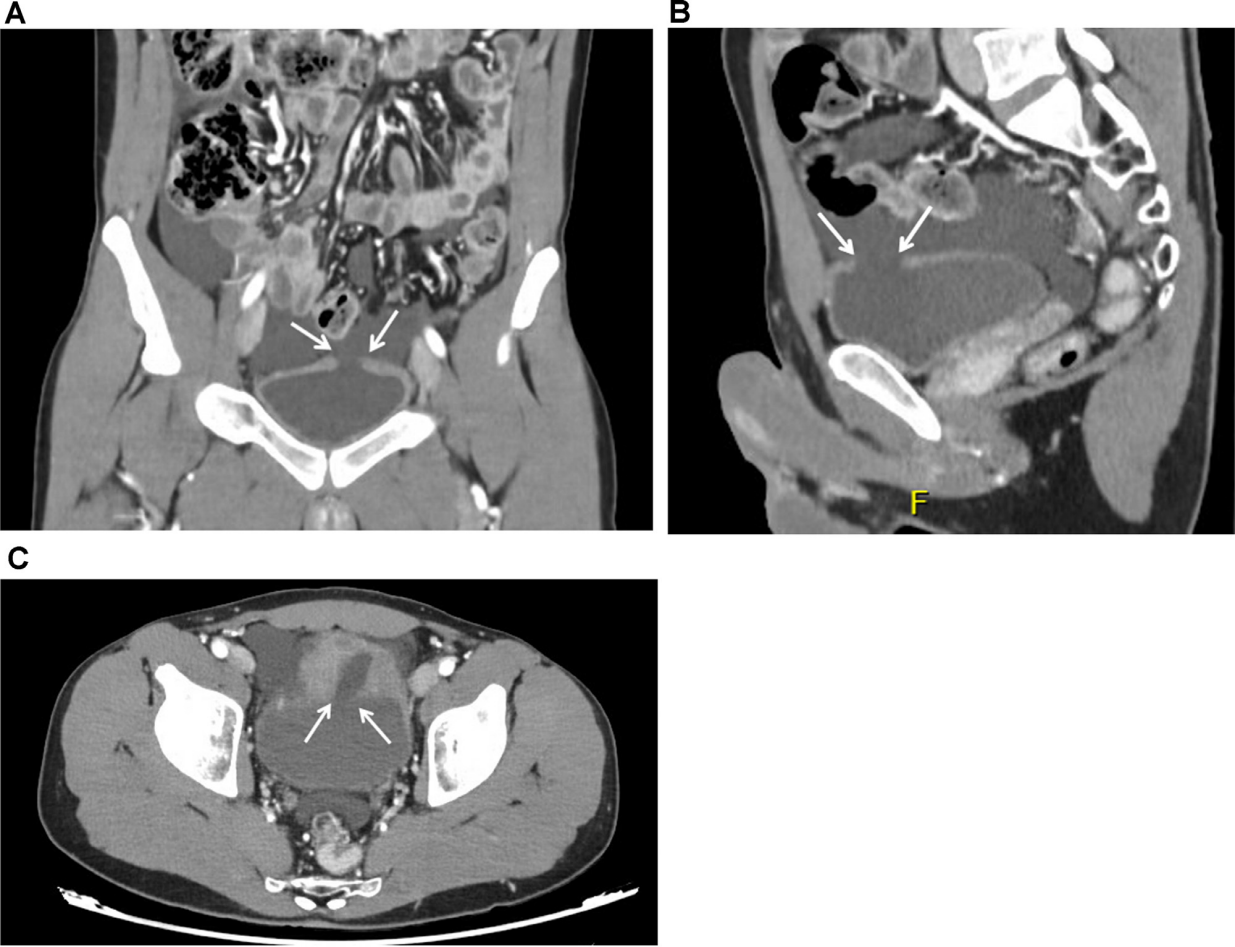
Contrast-enhanced CT of the small pelvis showing rupture of the dome wall of the urinary bladder (indicated by white arrows). Coronal (A), Sagittal (B), Axial (C).

A 26-year-old male with no significant prior medical history presented to the emergency department with acute, progressive abdominal pain, following a physical confrontation with a friend, while both were intoxicated with alcohol. The abdominal pain was accompanied by episodes of vomiting. On arrival, the patients vital signs were within normal limits: noninvasive blood pressure (NIBP) was 138/85 mmHg, pulse rate was 88 beats per minute, temperature was 36.5°Celsius, oxygen saturation was 99 % and respiratory rate was 16 breaths per minute. Physical examination revealed generalized abdominal tenderness, without signs of an acute abdomen. Laboratory findings showed normal Hb, CRP and electrolytes. However, leucocyte count was elevated (21.2 × 10^9^/l, normal range 4.5-11.0 × 10^9^/l), as was serum creatinine (152 µmol/l, normal range 61.9-114.9 µmol/l), with an eGFR of 48 ml/min/1.73m2 (normal range >90 mL/min/1.73m^2^). Focused assessment with sonography for trauma (FAST) exam identified free fluid in the lower abdomen. A contrast-enhanced computed tomography (CT) scan conducted 1 hour after presentation to the emergency department, revealed a 1 × 4 cm bladder rupture along with a large collection of intra-abdominal free fluid (Fig. 1). No other traumatic injuries were identified on imaging.

**Fig. 2:**
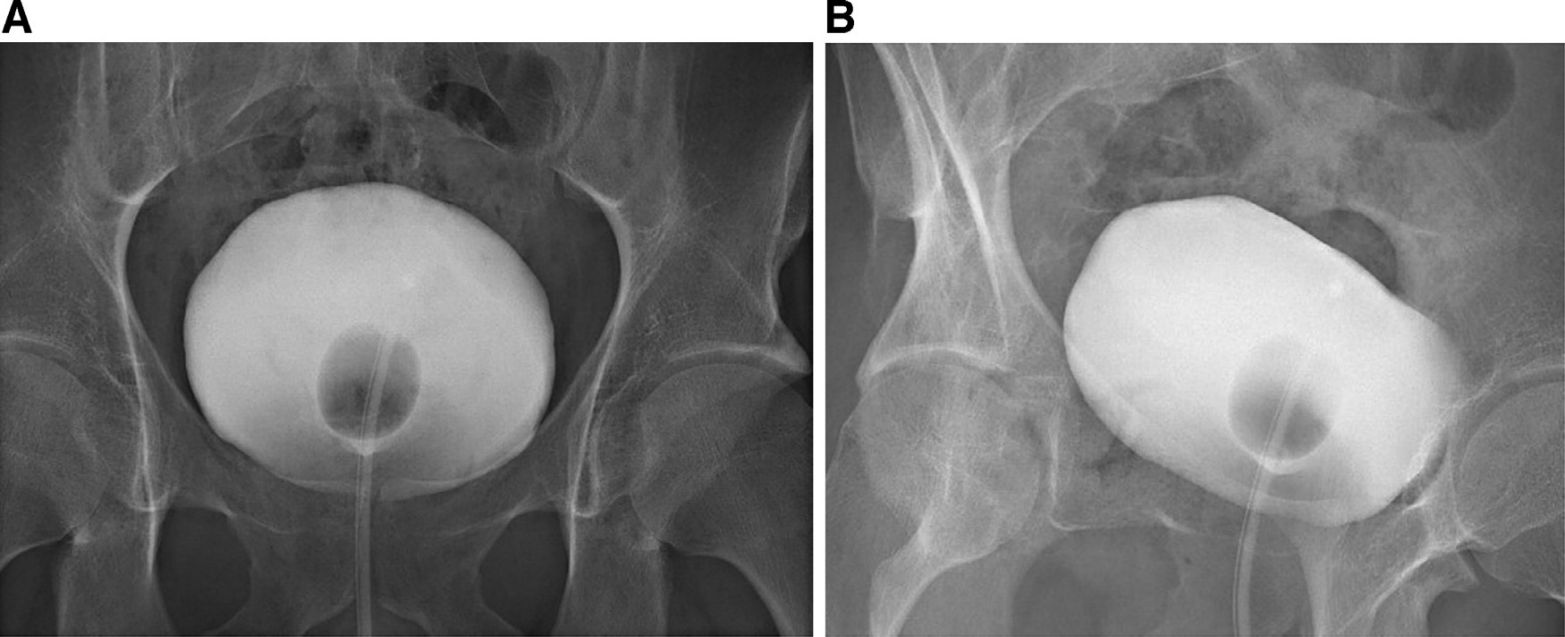
Cystography performed ten days after the bladder rupture. No urinary leakage was observed. Anteroposterior (A), Oblique (B).

A transurethral Foley catheter was placed and patient received broad spectrum antibiotics along with analgesics, which resulted in significant reduction of abdominal pain. The patient stayed in stable condition throughout admission. On the following day, an additional ultrasound was performed, which did not show any residual free fluid. The patient was discharged in stable condition 2 days postadmission. Ten days post-trauma, cystography revealed no evidence of urine leakage (Fig. 2). Subsequently, the Foley catheter was removed, and trial without catheter was successful and without any complications.

## Discussion

Bladder ruptures are rare but potentially life-threatening medical emergencies, that require rapid diagnosis and intervention. Most bladder ruptures are caused by high impact external traumas and accompanied by pelvic fractures.

This case presents a rare instance of an intraperitoneal bladder rupture following a seemingly low-impact altercation, which is particularly unusual in a young, otherwise healthy individual. Literature indicates that bladder ruptures may also occur following relatively low-impact traumas, especially when a full, distended bladder is present at time of trauma. Risk factors for bladder ruptures include radiotherapy, neurogenic bladder, recurrent cystitis, alcohol intoxication, urinary retention and bladder malignancies [2]. In our case, the patient presented with alcohol intoxication and possibly a full bladder at the time of trauma, which likely contributed to the vulnerability of the bladder dome to rupture.

Symptoms of bladder ruptures involve hematuria, reported in 77%-100% of cases, along with abdominal pain, nausea, vomiting and difficulty voiding. Other symptoms may depend on the mechanism of trauma [2]. Laboratory findings often reveal elevated serum creatinine levels due to reabsorption of urine through the peritoneal membrane. FAST exam can aid in detecting abdominal free fluid, but the preferred imaging modality for definitive diagnosis is a CT scan, ideally in combination with CT cystography for optimal visualization [8].

Urological trauma guidelines recommend surgical repair for intraperitoneal bladder ruptures to lower the risk of life-threatening complications, such as peritonitis, sepsis, and electrolyte disturbances [4,5]. Extraperitoneal bladder ruptures are managed conservatively, by placement of a transurethral Foley catheter. If necessary this is combined with percutaneous drainage. Follow-up cystography is recommended 7-10 days post-trauma before catheter removal to ensure full recovery [9]. Increasing evidence supports the feasibility of conservative management for select cases of intraperitoneal bladder ruptures [6,7,10].

In our case, conservative management of a large intraperitoneal bladder rupture was successful. We hypothesize that in our successful conservative treatment rapid diagnosis and treatment was essential. The patient presented to the emergency department within 30 minutes after trauma. One hour after presentation, the CT scan was completed, followed by immediate placement of a transurethral Foley catheter and intravenous administration of broad spectrum antibiotics. Moreover, the patient's young age and overall good health may have contributed to favorable tissue healing and spontaneous bladder repair.

The patient remained hemodynamically stable throughout the hospital stay and follow-up ultrasound performed 1 day postadmission showed no residual abdominal free fluid. If the patient had developed signs of generalized peritonitis, such as hemodynamic instability, increased pain or worsening of laboratory parameters, further CT imaging and surgical intervention would have been warranted. Our case highlights the importance of individualized patient assessment when deciding between conservative management or surgical repair for intraperitoneal bladder ruptures.

## Conclusion

Our case report demonstrates the successful conservative management of a significant intraperitoneal bladder rupture. Based on this case, we recommend considering conservative treatment for intraperitoneal bladder ruptures in patients with stable clinical conditions and no signs of peritonitis. Complete drainage of extravasated urine is critical for avoiding intervention and surgical intervention should be pursued.

## Patient consent

Informed consent was obtained from the patient for publication of this case report in accordance with the journals patient consent policy.

## Ethical approval

The study conforms to recognized standards of World Medical Association Declaration of Helsinki.

## Data availability

Data used and/or analyzed from the current study are available from the corresponding authors per request.

## CRediT authorship contribution statement

**Noah J. Sandel:** Conceptualization, Data curation, Investigation, Project administration, Methodology, Writing – original draft. **Matthijs Duijn:** Conceptualization, Resources, Writing – review & editing. **Liselotte M.S. Boevé:** Writing – review & editing, Supervision.
